# Cell Reprogramming, Transdifferentiation, and Dedifferentiation Approaches for Heart Repair

**DOI:** 10.3390/ijms26073063

**Published:** 2025-03-27

**Authors:** Micael Almeida, José M. Inácio, Carlos M. Vital, Madalena R. Rodrigues, Beatriz C. Araújo, José A. Belo

**Affiliations:** Stem Cells and Development Laboratory, iNOVA4Health, NOVA Medical School|Faculdade de Ciências Médicas, Universidade NOVA de Lisboa, 1169-056 Lisbon, Portugal; micael.almeida@nms.unl.pt (M.A.); carlos.vital@nms.unl.pt (C.M.V.); madalena.rodrigues@nms.unl.pt (M.R.R.); beatriz.araujo@nms.unl.pt (B.C.A.)

**Keywords:** direct reprogramming, indirect reprogramming, transdifferentiation, dedifferentiation, cardiovascular diseases

## Abstract

Cardiovascular disease (CVD) remains the leading cause of death globally, with myocardial infarction (MI) being a major contributor. The current therapeutic approaches are limited in effectively regenerating damaged cardiac tissue. Up-to-date strategies for heart regeneration/reconstitution aim at cardiac remodeling through repairing the damaged tissue with an external cell source or by stimulating the existing cells to proliferate and repopulate the compromised area. Cell reprogramming is addressed to this challenge as a promising solution, converting fibroblasts and other cell types into functional cardiomyocytes, either by reverting cells to a pluripotent state or by directly switching cell lineage. Several strategies such as gene editing and the application of miRNA and small molecules have been explored for their potential to enhance cardiac regeneration. Those strategies take advantage of cell plasticity by introducing reprogramming factors that regress cell maturity in vitro, allowing for their later differentiation and thus endorsing cell transplantation, or promote in situ cell proliferation, leveraged by scaffolds embedded with pro-regenerative factors promoting efficient heart restoration. Despite notable advancements, important challenges persist, including low reprogramming efficiency, cell maturation limitations, and safety concerns in clinical applications. Nonetheless, integrating these innovative approaches offers a promising alternative for restoring cardiac function and reducing the dependency on full heart transplants.

## 1. Introduction

Cardiovascular disease (CVD) is the leading cause of death in both developed and developing countries. According to the World Health Organization (WHO), 17.9 million people across the globe (31%) die due to CVD, of which 85% die from myocardial infarction (MI) [1].

MI results from a blockage in the coronary artery, depriving the heart muscle of oxygen and leading to ischemia. Without treatment, necrosis of cardiac muscle cells is inevitable, triggering electrical, metabolic, and contractile dysfunctions [2]. Upon cardiac lesion in adult mammals, the lost cardiomyocytes (CMs) are not fully replaced by new functional cells. Instead, unfavorable heart tissue remodeling (fibrotic scar tissue) occurs, leading to further functional loss [3]. It has been demonstrated that, despite being terminally differentiated, mature CMs can re-enter the cell cycle and divide, albeit at a lower rate compared to that in the neonatal period when they are still pre-mitotic [4,5]. Notably, approximately 50% of CMs are exchanged throughout the lifespan, indicating a continuous repopulation of these cells. Under normal physiological conditions in humans, it is estimated that about 1% of new adult CMs are generated annually. This regeneration rate declines with age but is likely to increase following injury [4,6,7,8]. Alternatively, this process can be influenced by manipulating the CM cell cycle, signaling pathways, endogenous gene expression, and environmental factors [4,5]. However, these interventions often lead to minimal myocyte renewal or cardiomegaly due to excessive activation of CM proliferation [4,5]. Therefore, the primary challenge in the cardiac regeneration field lies on identifying an optimal treatment strategy that can replenish the CM population without triggering uncontrolled proliferation and, at the same time, allow for the interaction between pre-existing and regenerated tissue, contributing to correct heart regeneration without causing heart failure (HF) [4,5,9].

The reprogramming mechanisms aim to induce differentiated cells to revert to a less differentiated state or even pluripotency. Their reacquired characteristics then allow them to replicate more efficiently or differentiate into almost any cell type [3,10]. Induced reprogramming to pluripotency seeks to induce differentiated cells to revert to pluripotency, generating stem cells that can differentiate into any intended cell type and then undergo tissue transplantation [11]. On the other hand, direct reprogramming is a progressive conversion that can occur in situ within cells, eliminating the need for external cell manipulation [3,12]. Depending on the factors administered, two critical processes could be triggered: transdifferentiation and dedifferentiation [3,13]. In transdifferentiation, mature somatic cells retrocede to a transitory and less differentiated state that allows them to switch lineages and differentiate into another cell type without going through an intermediate pluripotent state or progenitor cell type [3,13]. In dedifferentiation, terminally differentiated cells partially rewind within their own lineage, regaining the ability to proliferate and differentiate, ultimately replenishing the lost tissue [3]. Therefore, by leveraging both induced reprogramming to pluripotency and direct reprogramming of CM mechanisms, it becomes possible to repair the damaged tissue.

In this review, we explore topics aimed at restoring the integrity and function of the human heart, with the primary goal of achieving this without the need for heart transplantation.

## 2. Mechanisms of Cardiac Regeneration: From Neonatal Regenerative Capacity to Post-Infarction Repair

The neonatal heart possesses a remarkable ability to regenerate, a capability that, however, lasts until shortly after birth. During this period, CMs, which are still able to proliferate, exit the cell cycle and become mostly terminally differentiated [14,15]. During the neonatal period, heart cell proliferation is driven by factors such as GATA-binding factor 4 (GATA4), a transcription factor crucial for CM replication; Erb-b2 receptor tyrosine kinase (Erbb) 2 and bone morphogenetic protein (BMP) signaling also play key roles in regulating CM proliferation [4,6,7,8,14]. Additionally, inhibiting adrenergic receptor (AR) and thyroid hormone pathways may further enhance heart tissue regeneration [16].

**Figure 1:**
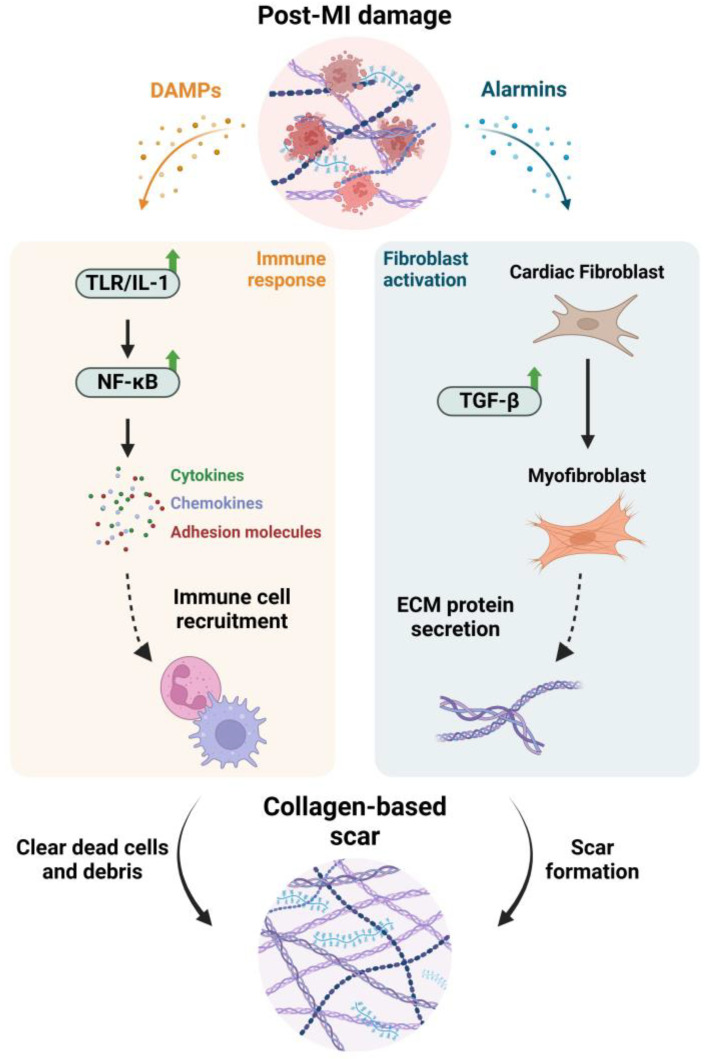
Innate mechanisms of response to myocardial infarction (MI). Following an MI, damaged cells and extracellular matrix (ECM) proteins release danger-associated molecular patterns (DAMPs) and alarmins, prompting an immune response and fibroblast activation, respectively. DAMPs will activate toll-like receptor/interleukin-1 (TLR/IL-1) signaling, triggering the nuclear factor (NF)-κB system and the subsequent release of chemokines, cytokines, and adhesion molecules. In response, immune cells will be recruited to the site of the lesion, clearing dead cells and debris. Concomitantly, alarmins will promote the conversion of fibroblasts into myofibroblasts, though the activation of the TGF-β pathway. Myofibroblasts will produce ECM proteins that will form a collagen-based scar, preventing cardiac rupture. Created with BioRender.com.

In adults, after an MI, an interplay of several processes, including inflammation, CM dedifferentiation and proliferation, and extracellular matrix turnover, results in the replacement of damaged heart muscle cells by scar tissue (Figure 1) [9,17,18,19,20].

This transformation process involves the activation of cardiac fibroblasts (CFs), which turn into myofibroblasts, leading to stiffness and scarring. This is associated with poor prognosis and HF [9,17,19,20]. Although these fibroblasts help maintain the heart’s structure post-MI, they also result in a non-contracting scar [9,17,18,19,20]. These occurrences lead to disease progression, impairing heart function, obstructing blood flow, reducing heart’s pumping ability, and causing further heart remodeling, ultimately resulting in HF [9,17,18,19,20].

Immediately after an MI, necrotic cells release alarmins that induce a pro-inflammatory and matrix-degrading fibroblast phenotype, facilitating leukocyte recruitment [21,22,23,24]. While dead cells and matrix debris are cleared, anti-inflammatory pathways are triggered, and transforming growth factor (TGF)-β cascades are activated, converting fibroblasts into α-smooth muscle actin (α-SMA)-expressing myofibroblasts [21,22,23,24,25]. These myofibroblasts secrete significant amounts of matrix proteins, forming a collagen-based scar that protects the infarcted region from complications like cardiac rupture [21,22,23,24].

Danger-associated molecular patterns (DAMPs) released from dying cells and damaged matrix components activate the complement cascade and toll-like receptor (TLR)/interleukin (IL)-1 signaling. This triggers the nuclear factor (NF)-κB system, leading to the production of chemokines, cytokines, and adhesion molecules [21,22,23,24]. As a result, neutrophils and mononuclear cells infiltrate the affected area, clearing dead cells and debris while stimulating reparative pathways [21,22,23,24].

Meanwhile, the immune response is crucial for repair, and the excessive production of reactive oxygen species (ROS) and the failure of the antioxidant system to neutralize them can lead to oxidative stress, further exacerbating inflammation and contributing to adverse remodeling of the infarcted heart [21,22,23,24,26]. The clearance of the infarcted area can be expedited by rapid angiogenic revascularization, which is essential for heart regeneration, preventing further complications [21,22,23,24]. Collectively, these findings suggest that activating the acute immune response through TLR signaling can enhance heart regeneration by promoting macrophage recruitment, neutrophil clearance, neovascularization, CM proliferation, and scar resolution [21,22,23,24].

The functional regeneration of an injured heart requires the clearance of necrotic tissue and the restoration of lost muscle [4,7,27]. This can occur through tissue reorganization, the activation of adult somatic stem cells, or cellular dedifferentiation and transdifferentiation [4,7,25,27]. Depending on the extent of the damage, these mechanisms may serve as critical cell sources for tissue replenishment, thereby contributing to overall cardiac integrity. Following this process, the blood supply is re-established, newly formed CMs undergo electrical integration, and inflammation is resolved, which culminates in the restoration of tissue structure and polarity [4,7,27].

However, these natural processes alone are insufficient for complete cardiac restoration, often leading to HF. Enhancing regenerative strategies, such as the transplantation of external cell patches, the administration of pro-regenerative factors (e.g., angiogenic agents), and the use of extracellular matrix scaffolds, may provide promising approaches for improving heart repair and function.

## 3. Induced Reprogramming to Pluripotency

Several types of stem cells hold immense potential for revolutionizing heart treatment, including skeletal myoblasts (SMs), bone-marrow-derived mononuclear cells (BMMNCs), hematopoietic stem cells (HSCs), endothelial progenitor cells (EPCs), mesenchymal stem cells (MSCs), cardiac stem cells (CSCs), embryonic stem cells (ESCs), and induced pluripotent stem cells (iPSCs) (Table 1). Among these, ESCs are particularly noteworthy due to their ability to grow indefinitely while maintaining pluripotency, meaning that they can differentiate into cells from all three germ layers. This makes them valuable tools for cardiac regeneration, modeling molecular mechanisms associated with cardiovascular diseases (CVDs), and identifying new drugs. However, ethical concerns arise because ESCs are derived from blastocysts [6]. Additionally, these cells pose significant immune compatibility challenges when transplanted into patients [6].

To address these concerns, researchers have explored the induced reprogramming to pluripotency of adult somatic cells, generating pluripotent cells directly from the patient’s own cells. In 2006, Japanese scientists introduced four transcription factors—octamer-binding transcription factor (Oct) 3/4, SRY-box transcription factor (Sox) 2, c-Myc, and Kruppel-like factor 4 (Klf4)—into murine fibroblasts using retroviruses, leading to the creation of the first iPSCs, named for their functional similarities to ESCs [28,29]. Similarly, in 2007, hiPSCs were generated from adult human dermal fibroblasts using the same Yamanaka factors (human Oct3/4, Sox2, Klf4, and c-Myc) [30]. Today, this technology is significantly more accessible, with several high-quality iPSC lines banked and readily available for research purposes.

The iPSC reprogramming process involves several key steps, including alkaline phosphatase activation, the silencing of somatic-specific gene expression, the upregulation of stage-specific embryonic antigen (SSEA) 1, and the gradual silencing of exogenous genes while increasing the expression of endogenous pluripotency markers such as Oct4 and Nanog [17,30,31,32]. These cells can be derived from various sources, with adult fibroblasts, peripheral blood mononuclear cells, and epithelial cell types being commonly used for reprogramming. Importantly, iPSCs retain genetic and immunological characteristics related to their original somatic cell lineage [33]. Although they undergo reprogramming, their tissue memory influences genetic and epigenetic features, such as DNA methylation and histone modification. This can impact their phenotypic behavior and major histocompatibility complex (MHC) presentation, which varies depending on the cell type of origin and the time elapsed since cell collection [33]. These factors are particularly relevant when considering autologous transplantation, as they can affect immune compatibility and therapeutic efficacy [33].

**Table 1:** Pluripotent and tissue stem cells for cardiovascular therapy: characteristics, advantages, and clinical applications.

Cell Type	Description	Advantages	Disadvantages	Use in Clinical Trials
BMMNCs [6,34,35]	Mixed population containing HSCs and MSCs	Abundant, easily accessible, autologous transplantation	Limited differentiation potential	Improved myocardial performance after transplantation
HSCs [6,36,37]	Subpopulation of BMMNCs, can differentiate into all blood cells	Autologous transplantation, well-established isolation protocols	Limited abundance, weak differentiation potential	Limited therapeutic use
EPCs [6,38]	Can differentiate into endothelial cells, promote blood vessel growth	Homing capacity, paracrine signaling stimulates endothelial proliferation	Reduced differentiation capacity	Shown to improve heart function in some studies
MSCs [6,35,39]	Immunomodulatory, anti-inflammatory properties	Improve cardiac function, reduce inflammation Direct effect on cardiac tissue repair and regeneration The secretion of trophic factors improves cardiac function by tissue injury reduction, inhibition of fibrotic remodeling, angiogenesis, and activation of host tissue stem cell niches	Conflicting results in clinical trials	Most studied stem cells for cardiac injury
CSCs [6,35,40]	Isolated from heart tissue, can differentiate into CM	Preserve heart function, stimulate angiogenesis	Limited number of cells	Shown to improve heart function in animal models
ESCs [35,41]	Pluripotent, can differentiate into any cell type	High differentiation potential, self-renewal capacity	Ethical concerns, poor engraftment, risk of teratoma formation	Not used due to ethical and safety concerns
iPSCs [41]	Generated from adult cells, can differentiate into any cell type	Autologous transplantation, no ethical concerns, self-renewal capacity	Immature cells, risk of tumorigenicity, low numbers of pure and mature CMs	Shown to improve cardiac function in animal models

### Advances in Pluripotent Cell Differentiation into Functional Cardiac Cells

Cardiac reprogramming using transcription factors, miRNAs, or small molecules holds promise for repairing damaged heart tissue. Several approaches are being explored, including stimulating heart muscle cells to proliferate and transplanting cardiac progenitor cells (CPCs) or CMs derived from iPSCs [42,43,44,45,46,47].

**Figure 2:**
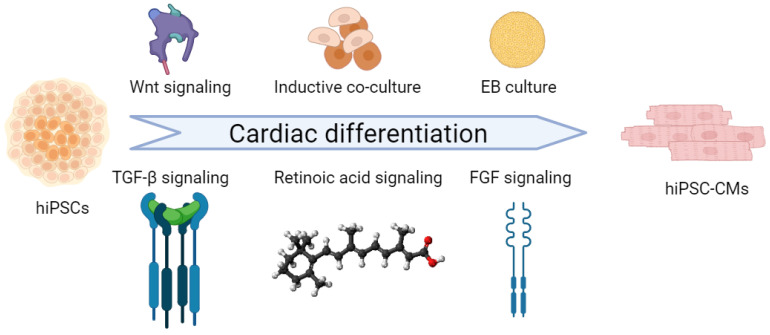
hiPSC-derived cardiomyocyte generation. Cardiac differentiation of hiPSCs through the modulation of Wnt, TGF-β, FGF, and RA signaling pathways. Inductive co-culture and embryoid body formation are employed in cardiac research to improve the physiological and metabolic relevance of in vitro hiPSC-derived CMs. Created with BioRender.com.

To generate iPSC-derived CMs, researchers have developed efficient protocols using monolayer cultures, inductive co-culture, and the spin embryoid body (spin-EB) method. These techniques employ small molecules to target specific developmental signaling pathways, including WNT, activin/nodal, BMP, retinoic acid (RA), fibroblast growth factor (FGF), and Notch (Figure 2). Key downstream effectors such as t-box transcription factors (TBX1/5), forkhead box F1 (FOXF1), heart- and neural crest-derived transcript (HAND1/2), TBX2/3, LIM homeodomain protein (ISL1), Iroquois homeobox 4 (IRX4), Hes related family BHLH transcription factor with YRPW motif 1/2(HEY1/2), and nuclear receptor subfamily 2 group F member (NR2F1/2) guide progenitor specification, morphogenesis, and physiological maturation at specific developmental stages, ultimately leading to CM generation [48,49,50,51,52].

The differentiation process consists of three distinct stages. In the early stage of mesodermal induction, bioactive lipids such as sphingosine-1-phosphate (S1P) and lysophosphatidic acid (LPA) enhance CM generation by increasing mediators that promote the nuclear accumulation of β-catenin and activate the Wnt signaling pathway. This is followed by the activation of signaling pathways, including those associated with BMPs and FGFs, that contribute to CPC formation, while Wnts, TGF-β/activin/nodal, and Notch play crucial roles at multiple stages—inducing mesoderm formation, specifying the cardiac lineage, maintaining cardiac progenitors, and facilitating the final maturation of CMs. Still, methods for sub-type differentiation are being explored to generate atrial or ventricular CMs, with RA being a key factor in this process [48,53,54].

iPSC-derived CM differentiation has evolved significantly in recent years, although the current methods still face limitations in CM maturation [31]. Notably, iPSC-derived CMs tend to resemble cells in an immature stage in terms of marker expression, ultrastructural features, metabolic signature, and electrophysiological properties, which hinders their in vivo applications [17,28,31,32,52,55,56].

Although still not clear, the origin of somatic cells may be a critical factor in stem cell research, as conflicting results on its influence on iPSC-derived CM differentiation and maturation have been reported [17,33,57,58,59,60]. iPSCs derived from somatic cells of cardiac origin exhibit an enhanced capacity for cardiac re-differentiation due to the upregulation of key cardiac genes, including myosin heavy chain (MYH6), troponin I (TNNI3), potassium voltage-gated channel subfamily Q member 1 (KCNQ1), and potassium voltage-gated channel subfamily E regulatory subunit 1 (KCNE1). Additionally, the effectiveness of iPSC-derived CMs is highly dependent on maturation and purification processes, as defective cells can develop during in vitro culture. To address this issue, distinct metabolic flow-based purification technologies have been designed for large-scale applications, utilizing sequential glucose depletion, lactate selection, and fatty acid-based metabolic shifts [17,61,62].

Despite these scientific advances, several technical challenges remain. Nevertheless, the application of these novel therapeutical approaches in human patients must rely on strict ethical regulations that may still be underdeveloped due to the novelty of the field. Adherence to regulations and guidelines must be continuously refined and adapted to ensure the ethical advancement of iPSC-derived CM research and applications [17,63,64].

## 4. Direct Cell Reprogramming into Cardiomyocytes

**Figure 3:**
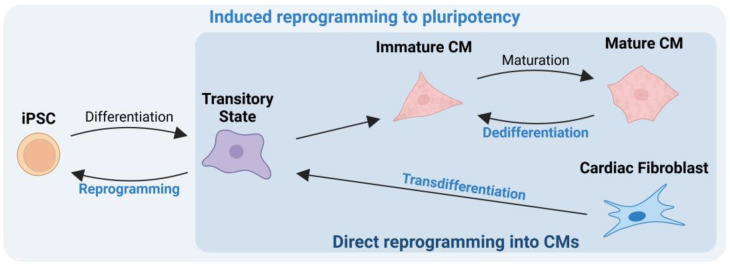
Direct cell reprogramming into cardiomyocytes (CMs). Induced reprogramming to pluripotency involves the complete regression of cell differentiation into a pluripotent status (iPSC), allowing for further differentiation into a cell type from a different cell lineage. On the other hand, direct cell reprogramming, encompassing transdifferentiation and dedifferentiation, does not require the intermediate step of generating a pluripotent cell type. Transdifferentiation involves the regression of a somatic cell to a less differentiated transitory state from where the cell may develop into a different lineage. Dedifferentiation consists in the generation of a less differentiated (more immature) cell type, in the same lineage of the initial somatic cell. Created with BioRender.com.

Direct cell reprogramming techniques offer several advantages over other cell reprogramming methods, including simplicity, standardization, and versatility [17,65]. Additionally, these patient-specific therapies reduce ethical and safety concerns. The human heart consists of approximately 30% CMs and 60–70% cardiac fibroblasts (CFs) [66]. Delivery methods can specifically administer reprogramming factors to convert scar-forming CFs into new heart muscle cells, known as induced cardiomyocytes (iCMs), or induce an immature state in CMs. These reprogrammed cells can proliferate and repopulate the damaged area (Figure 3). However, the possibility of reprogramming off-scar cells must also be considered [17,42,53,67].

This approach offers several advantages, particularly for in vivo repair. Administering reprogramming factors directly into the damaged heart eliminates the need for complex cell manipulation techniques and reduces the risk of tumor formation by bypassing the intermediate pluripotent stem cell (PSC) stage, thereby lowering the tumorigenic potential compared to other methods [17,42,53,67].

Studies in mice have demonstrated that in vivo reprogramming can enhance heart function following MI. Reprogramming CFs into iCMs reduces scar tissue formation and improves overall cardiac health. Tracing studies confirmed that these newly generated iCMs arise directly from CFs rather than from fusion with pre-existing heart muscle cells. Furthermore, iCMs produced in vivo more closely resemble natural cardiomyocytes than those generated through in vitro methods [17,67]

Several signaling pathways, including TGF-β, Wnt, Notch, and Akt serine/threonine kinase (Akt) pathways, interact to influence cell differentiation [17,67]. For example, inhibiting TGF-β and Wnt signaling has been shown to enhance cardiac reprogramming efficiency and maturation. iCMs reprogrammed using the TGF-β inhibitor SB431542 exhibit the downregulation of genes associated with fibrotic signaling and extracellular matrix formation [68,69]. Similarly, iCMs reprogrammed with the Wnt inhibitor XAV939 show reduced expression of genes involved in chromatin modulation, DNA packaging, and nucleosome organization and, thereby, improved transcription factor accessibility [70].

Reprogramming CFs into iCMs relies on specific combinations of transcription factors, such as Gata4, Hand2, myocyte enhancer factor 2C (Mef2c), mesoderm posterior protein (Mesp1), and NK2 homeobox 5 (Nkx2.5) [71,72]. Another promising strategy involves miRNAs, which regulate multiple signaling pathways simultaneously to enhance reprogramming. Molecules such as miR-1, miR-133, miR-208, and miR-499 target and suppress genes that maintain fibroblast identity, thereby promoting the transition to a cardiac phenotype [73,74].

Despite its potential, direct reprogramming into cardiomyocytes faces several challenges. The current methods have low efficiency, requiring improvements in the CF-to-iCM conversion rate and better strategies for ensuring a stable cell fate. Additionally, the delivery of reprogramming factors—whether via viral vectors or lipid-based transfection—remains a challenge, as these methods can introduce unintended changes in the host’s DNA [17,67,71,72]. Further advancements in delivery techniques and reprogramming efficiency are necessary to make this approach a viable clinical therapy [17,67,71,72].

### 4.1. Transdifferentiation to Generate Cardiac Cells

**Figure 4:**
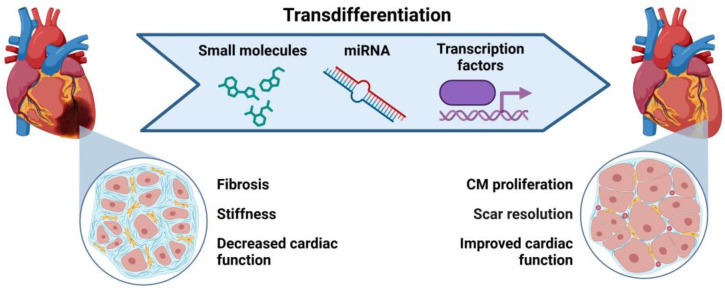
Cardiac fibroblast transdifferentiation into cardiomyocytes (CMs). Small molecules, miRNAs, and transcription factors are administered to induce the transition of cardiac fibroblasts (CFs) to induced cardiomyocytes (iCMs), leading to improved cardiac function and the resolution of excessive scar formation. Created with BioRender.com.

Transdifferentiation has gained significant attention in recent studies [42,53,73,75,76]. The direct reprogramming of cells—specifically, converting CFs into iCMs or induced cardiac progenitor cells (iCPCs)—can be achieved using transcription factors, miRNAs, and small molecules. This approach enables the direct conversion of non-myocytes into functional CMs in situ, eliminating the need for ex vivo cell transplantation and reducing the risk associated with delivering potentially oncogenic factors to stimulate cardiac proliferation (Figure 4). As a result, transdifferentiation offers a promising alternative cell source for heart regenerative medicine and cardiac transplantation [42,53,73,75,76].

Pioneering research identified a combination of three transcription factors—Gata4, Mef2c, and Tbx5 (GMT)—as a key regulator of gene expression during embryogenesis and cellular reprogramming. These factors can successfully convert fibroblasts into iCMs in the mouse by essentially rewinding the cell’s identity to a CM state. The expression levels and relative ratios of these three genes determine the reprogramming trajectory, guiding cells through intermediate fibroblast, pre-iCM, and iCM states [43,71,77,78,79,80].

Further studies have highlighted additional transcription factors, such as Hand2 (GHMT), Nkx2.5, myocardin (MYOCD), serum response factor (SRF), Mesp1, BRG1/BRM-associated factor 60C (BAF60C), SWI/SNF-related matrix-associated actin-dependent regulator of chromatin subfamily D member 3 (SMARCD3), and spalt-like transcription factor 4 (Sall4), which enhance GMT’s cardio-inducing effect [43,80,81,82,83,84]. However, while many genes contribute to transdifferentiation, manipulating all these factors simultaneously could compromise cell viability, making it essential to identify the key factors necessary for efficient reprogramming.

Significant differences exist between mouse and human cardiac transdifferentiation, affecting iCM generation efficiency. Studies indicate that the conversion rates in human fibroblasts are lower than in mouse cells, and human iCMs exhibit slower maturation [85,86]. Additionally, human cells appear less plastic and have a higher epigenetic barrier to reprogramming compared to mouse cells. These differences become evident when examining the gene expression profiles during reprogramming, including markers such as vimentin, beta-III tubulin, and alpha-fetoprotein [87].

Further evidence suggests that TGF-β pathway modulation affects species-specific transdifferentiation efficiency. Inhibiting TGF-β enhances cardiac transdifferentiation in mouse cells, whereas TGF-β activation is beneficial for human fibroblast conversion [88].

In human CFs, GMT expression alone appears insufficient to induce transdifferentiation, at least in vitro. A more effective approach involves combining GMT with estrogen-related receptor gamma (ESRRG) and Mesp1, which successfully induces global cardiac gene expression and phenotypic shifts in human fibroblasts derived from ESCs, fetal heart tissue, and neonatal skin. Adding MYOCD and zinc finger protein/FOG family member 2 (ZFPM2) further enhances reprogramming by promoting sarcomere formation, calcium transients, and action potentials [89,90].

Additionally, the transduction of GMT, Mesp1, and Myocd (GMTMM) significantly upregulates a panel of cardiac genes related to sarcomere structure, ion channels, and transcription factors, compared to that of GMT alone [90]. Gene expression analysis during reprogramming also revealed the downregulation of mRNA processing and splicing factors. One notable barrier to iCM maturation is polypyrimidine tract binding protein 1 (Ptbp1), which regulates alternative splicing. The downregulation of Ptbp1 is critical for acquiring cardiomyocyte-specific splicing patterns [79].

miRNAs play a crucial role in cardiac reprogramming by regulating multiple signaling pathways simultaneously. A combination of four miRNAs—miR-1, miR-133, miR-208, and miR-499—has been shown to enhance the reprogramming efficiency when delivered via Dharmafect1 (a commercial lipid transfection product). In vitro, these miRNAs induce cardiomyocyte gene expression in human CFs, while in vivo, they improve the reprogramming efficiency in adult mice subjected to left anterior descending coronary artery ligation [74].

Further studies revealed that combining miR-133 with GMT or MESP1 and MYOCD with GMT enhanced cardiac reprogramming in mice by inhibiting Snai1 and suppressing the fibroblast phenotype [91]. Another promising approach involves miR-2392, which regulates cell growth and differentiation via mitogen-activated kinase-like protein (MAPK) and Wnt signaling pathways. When cloned into a lentiviral vector and delivered to human dermal fibroblasts, followed by treatment with forskolin, valproic acid, and CHIR99021, this strategy modestly induced a CM-like phenotype [92].

Additionally, Zhou et al. demonstrated that the optimal ratio of reprogramming factors is critical for human cardiac reprogramming. Using MEF2C, GATA4, and TBX5 (MGT) combined with miR-133 significantly increased cardiac troponin T (cTnT) expression compared to other methods. However, a superior conversion rate was achieved with GATA4, HAND2, MEF2C, TBX5, MYOCD, MESP1, miR-1, and miR-133 (GMTMyMsp-miR-1/133 cocktail) [93,94,95].

Recent advances suggest that activating endogenous cardiac genes via CRISPR-based gene activation can significantly enhance reprogramming. Activation of Gata4, Nkx2.5, Tbx5, and Hand2 in adult extracardiac fibroblasts using a CRISPR activation system generated ~80% of CRISPR-induced cardiac progenitor cells (ciCPCs) in vitro. When transplanted in vivo, ciCPCs differentiated into cardiovascular cells, improved the contractile function, and reduced scar formation [71,96,97].

Additionally, Li et al. improved the differentiation process of GHMT transdifferentiation by incorporating three cardioinductive growth factors—BMP4, activin A, and bFGF—which efficiently induced over 80% of protein-induced CPCs from human dermal fibroblasts, downregulating fibroblast markers while upregulating cardiac progenitor-specific markers [71].

Epigenetic modifications, such as histone methylation, phosphorylation, acetylation, and ubiquitylation, play a significant role in regulating gene expression during reprogramming. CM-specific loci bound by Bmi1 in fibroblasts are marked by the H2AK119Ub modification, and these regions also contain binding sites for Ring1B and enhancer of zeste homolog 2 (Ezh2)—two chromatin remodelers that enhance iCM reprogramming efficiency and are conserved in both mice and humans [75,98,99].

#### Small Molecules in Cardiac Transdifferentiation

Genetic manipulation raises safety concerns, which makes it undesirable for most clinical applications. Therefore, the discovery of chemical cocktails (Table 2) capable of inducing reprogramming features suggests the possibility of replacing certain previously described genetic elements. It has been reported that small chemical molecules can enhance the efficiency, speed, and quality of iCM generation, both in vivo and in vitro, by downregulating fibroblast gene expression and activating cardiac gene expression in mouse and human models [51,68,77,91,100,101,102,103,104,105,106].

TGF-β inhibitors facilitate mesenchymal-to-epithelial transition (MET), a crucial step in the early stages of cellular reprogramming, by suppressing Smad2 and 3 phosphorylation [68,69,107]. SB431542, for instance, can substitute Oct4 during reprogramming, and OAC2 is an Oct4-activating compound that promotes gene expression through the Oct4 gene promoter [108,109,110]. RepSox can replace Sox2 in fibroblast reprogramming, while A83-01 targets the activin/NODAL/TGF-β pathway by inhibiting ALK5, ALK4, and ALK7 [111,112].

The Wnt pathway regulates differentiation into mesoendodermal lineages. CHIR99021, an inhibitor of glycogen synthase kinase 3 beta (GSK3β), activates the Wnt pathway, while XAV939 inhibits tankyrase (TNKS) 1 and TNKS2, thereby suppressing them [102,113].

MAPK/extracellular signal-regulated kinase (ERK) signaling regulates c-Myc and hypoxia-inducible factor-1α (HIF-1α), both of which act downstream of MAPK/ERK. This pathway is critical for metabolic reprogramming, shifting the metabolism from oxidative phosphorylation to glycolysis, and is also important for cell cycle re-entry in postmitotic muscle cells during regeneration [114]. PD0325901 inhibits MEK activation and its downstream signaling, thereby suppressing ERK phosphorylation [115,116]. Forskolin functions as an activator of the cyclic adenosine monophosphate (cAMP) pathway, while SC1 is a dual phosphatidylinositol 3-kinase (PI3K) and MEK/ERK pathway inhibitor that targets Ras-specific GTPase-activating protein (RasGAP) and ERK1 [91,101,117].

RhoA-ROCK and TGF-β signaling are associated with pro-fibrotic events. The inhibition of these pathways enhances the ability of cardiogenic factors to reprogram mouse fetal and adult fibroblast into CMs [69]. RhoA-ROCK inhibition is enhanced through the suppression of SRF signaling [69,102]. Y-27632 reduces the expression of fibronectin containing extra domain A (Fn-EDA) and αSMA but does not change Snail and Slug expression in the context of cardiac reprogramming [69,102].

Insulin-like growth factor (IGF)1, PI3K, and Akt1 signaling promote a more mature cardiac phenotype in iCMs by activating mTORC1 and Foxo3a in embryonic fibroblasts; however, this is not observed in adult fibroblasts [102,118]. This pathway also functions through p38/MAPK activation [102,118,119]. Fibroblast growth factor 10 (FGF10) and vascular endothelial growth factor (VEGF) enhance chromatin accessibility and strengthen Mef2c binding to its genomic targets via p38/MAPK activation [118,120]. Additionally, SU16F and JNJ10198409, two inhibitors of the platelet-derived growth factor (PDGF) pathway, increase chromatin accessibility in cardiac regulatory regions [105].

Leukemia inhibitory factor (LIF) activates cardiac stem and precursor cells, influencing their proliferation through the Janus kinase (JAK), signal transducer and activator of transcription (STAT), MEK, ERK, and PI3K-Akt pathways [121].

RA affects gene expression in endothelial cells, smooth muscle cells (SMCs), CFs, and CMs, being observed to inhibit the proliferation of human aortic SMCs and murine CFs and, in the same way, promote differentiation in CPCs through the expression of cardiac-specific genes [122,123,124]. In PSCs, RA modulation guides the specification of heart field-specific progenitors, namely, its absence leads to mesoderm induction [123,125]. The RA receptor agonist TTNPB, in combination with other known reprogramming factors (C, CHIR99021; R, RepSox; F, forskolin; V, valproic acid (VPA); P, Parnate; T, TTNPB; M, rolipram, i.e., the CRFVPTM combination) facilitates the conversion of CFs into iCMs [126]. VPA, forskolin and rolipram have been reported to protect CMs and prevent collagen formation, reducing the fibrotic environment and improving factor accessibility and the consequent recovery of the infarcted area.

DNA accessibility is an essential factor for enhancing reprogramming efficiency. Histone demethylation and deacetylation compromise the efforts to activate important genes in reprogramming events [127]. AS8351, a histone demethylase inhibitor targeting KDM5B (JARID1B), is a component of a chemical cocktail that efficiently induces the cardiac reprogramming of human fibroblasts into iCMs [105]. Histone deacetylation inhibitors promote transcription by preventing chromatin condensation and transcriptional silencing, which are typically induced by these enzymes that remove acetyl groups [128].

VPA functions as a chromatin-remodeling enzyme inhibitor and promotes histone acetylation, opening or closing the chromatin structure [100]. Parnate, a lysine-specific demethylase 1 (LSD1) inhibitor, may act by inhibiting H3K4 demethylation, thereby enhancing the initial epigenetic activation of fibroblasts [129]. BIX-01294 is a histone methylation modulator and an inhibitor of G9a histone methyltransferase, which inhibits GLP and reduces H3K9me2 levels in bulk histones [127]. Additionally, it inhibits H3K36 methylation by NSD1, NSD2, and NSD3, thereby improving DNA accessibility [127].

**Table 2:** Suggested small molecules to promote somatic cell transdifferentiation.

Pathway/Function	Compound	References
TGF-β signaling inhibitor	SB431542	[68,101]
	RepSox	[91]
	A83-01 (TGF-type I receptor)	[105]
	OAC2	[105]
Wnt signaling activator	CHIR99021	[68,91,105]
	XAV939	[68]
MAPK/ERK signaling inhibitor	PD0325901	[91]
	Forskolin	[91,101]
	SC1	[105]
Rho-associated kinase (ROCK) pathway inhibitor	Y-27632	[105]
IGF1/PI3K/Akt1 signaling pathway activator	FGF10	[119]
	VEGF	[119]
	SU16F (platelet-derived growth factor receptor (PDGFR) inhibitor)	[105]
	JNJ10198409	[105]
Leukemia inhibitory factor	LIF	[91]
RA receptor agonist	TTNPB	[91]
DNA methylation inhibitor	AS8351	[105]
Histone deacetylation inhibitor	VPA	[91,100]
	Parnate	[91]
Histone methylation modulator	BIX-01294	[68,105]

### 4.2. Delivery Methods for Reprogramming Factors

Delivering GMT factors directly into cardiac fibroblasts is crucial for transdifferentiation [17]. Viral vectors are commonly used to efficiently deliver reprogramming factors, cell-derived protein factors, exosomes, and miRNAs to target cells [17]. However, they raise safety concerns due to their potential immunogenicity and the risk of insertional mutagenesis [17]. Several viral vectors have been explored, including adenoviruses (ADs), Sendai virus (SeV), lentiviruses, and adeno-associated viruses (AAVs) [80,94,130,131]. ADs and AAVs are particularly advantageous, as they can infect non-dividing cells [131]. However, AD vectors often trigger immune responses, which ultimately limits their efficacy in gene delivery [131]. In contrast, AAVs have lower immunogenicity and have been optimized for cardiac targeting to reduce off-target effects [74,132,133,134,135]. Among the AAV serotypes, AAV6 and AAV9 have demonstrated higher transduction efficiency in cardiac cells [17,74,132,133,134,135,136]. However, further improvements are needed to enhance their tropism towards heart cells before their widespread clinical application, as these vectors have not been optimized for human use due to ethical concerns [137].

Non-viral methods provide a safer alternative but typically exhibit lower efficiency compared to viral vectors [71,138,139,140,141,142]. Nanoparticles and extracellular vesicles (EVs) have been engineered as microscopic carriers for delivering reprogramming factors specifically to heart cells, with the potential to convert the recipient cells into CMs [71,138,139,140,141,142]. Nanoparticle-based delivery for CVDs is minimally invasive and has been investigated for the transport of therapeutic factors for several years [140,142,143,144,145]. Recent studies have supplied proof of concept for biomimetic and sequentially targeted nanomedicine in miRNA-mediated reprogramming therapy [140,142,143,144,145]. These delivery systems effectively reprogram fibroblasts into iCMs, both in vitro and in vivo, without cytotoxicity, leading to improved cardiac function and reduced fibrosis [140,142,143,145]. EV-based delivery is a rapidly growing field due to its advantages, including the absence of tumorigenicity, low immunogenicity, and demonstrated stability and efficacy in cardiac repair in clinical trials [145,146,147].

Advancements in the field should integrate previous research on direct drug delivery to the heart with emerging innovations in particle engineering. The continued optimization of both viral and non-viral delivery systems will be essential for enhancing specificity, efficiency, and safety in cardiac reprogramming therapies.

### 4.3. Challenges and Future Directions in Transdifferentiation

In an injured heart, the conversion of CFs into myofibroblasts exacerbates cardiac fibrosis, worsening the damage before the healing process begins [53,148]. The direct reprogramming to cardiomyocytes offers a promising strategy to mitigate this risk by using transcription factors or small chemical molecules to convert CFs into CMs without the need of an intermediate step for pluripotent cell generation, characteristic of approaches based on induced reprogramming to pluripotency [53]. This method not only reduces the risk of tumor formation but also expands the range of available cell sources, enhancing the potential for autologous cell transplantation in heart disease treatment [53]. By leveraging these techniques, the concerns regarding immune rejection can be minimized, enabling more effective and personalized therapies for cardiac regeneration [53].

Ongoing research is focused on refining targeted delivery techniques and optimizing combinations of reprogramming factors to induce specific CM subtypes from CFs [149,150]. However, the number of available CFs in the injured heart is limited [149,150]. To achieve complete functional restoration, an external cell source may be required. Ideally, this source should be an easily accessible and expandable sample that can be transdifferentiated in vitro under controlled conditions before transplantation [149,150]. For instance, bioengineering scaffolds designed to guide the organization and maturation of newly generated iCMs should be developed. These scaffolds can help ensure the proper integration of iCMs into the existing heart tissue, thereby facilitating functional restoration.

While transdifferentiation holds great promise, addressing its challenges is critical for its clinical application. Enhancing the reprogramming efficiency is paramount, requiring the optimization of both the reprogramming process and delivery methods to overcome the current limitations. Additionally, ensuring the safety and efficacy of transdifferentiation both in vivo and in vitro is essential and requires a rigorous evaluation in living organisms. A key aspect of successful cardiac reprogramming is a precise control over the location and type of the CMs generated. Controlled reprogramming strategies that target specific cardiac regions and ensure the generation of functionally appropriate CMs will be crucial for restoring optimal heart function.

### 4.4. Dedifferentiation for Cardiac Repair

Dedifferentiation and transdifferentiation share several similarities, with some transdifferentiation models requiring an initial dedifferentiation step [151]. Dedifferentiation is the process by which cells revert from a partially or terminally differentiated stage to a less differentiated stage within their own lineage [151]. A transient regenerative potential has been observed in neonatal murine hearts, attributed to the dedifferentiation of CMs [152,153]. It is hypothesized that CM remodeling is closely linked to dedifferentiation, which involves a shift in gene expression from a mature CM state to a more immature state [153,154]. This transition enables CMs to survive under unfavorable conditions, re-enter the cell cycle, proliferate, and recover their lost function [151,153,155,156].

Key pathways involved in the process of dedifferentiation include kynurenine and the aryl hydrocarbon receptor (AHR)–proto-oncogene tyrosine-protein kinase Src (SRC)–yes-associated protein (YAP)/ERK pathway [157]. Kynurenine has been shown to stimulate CM proliferation and angiogenesis via the AHR-SRC-YAP/ERK pathway [157]. Another crucial factor in this process is the inflammatory cytokine oncostatin M (OSM) that activates the Ras/mitogen-activated protein kinase (MEK)/ERK pathway [158,159,160]. This signaling cascade drives mature CMs into a more primitive state, allowing them to re-enter the cell cycle and proliferate. A positive feedback loop involving YAP, transcriptional enhancer factor TEA domain family member (TEAD)1, and OSM mediates CM dedifferentiation, with YAP-TEAD1 stimulating OSM expression, which in turn activates YAP-TEAD1 and upregulates OSM receptors [158,159,160]. The retinoblastoma (RB) protein is another vital critical regulator of dedifferentiation, enabling differentiated cells to re-enter the cell cycle and contribute to regeneration [161]. Unexpectedly, miR-1 and miR-133a negatively regulate CM proliferation [162,163,164]. Their temporary deletion has been shown to protect against MI by suppressing OSM receptor (OSMR) and fibroblast growth factor receptor 1 (FGFR1), while simultaneously activating the MEK/ERK pathway [162,163,164]. These two miRNAs present a contradiction in the possible preliminary dedifferentiation step in a transdifferentiation process.

The Hippo pathway, when inactivated, increases CM cell cycle activity [165]. YAP and tafazzin (TAZ), key transcription co-activators of the Hippo pathway, promote CM proliferation [9,165,166,167,168]. During embryonic development, Hippo pathway inactivation results in an enlarged heart due to heightened CM proliferation, whereas YAP deletion causes cardiac hypoplasia [169]. Conversely, constitutive YAP activation increases the myocardial mass through enhanced proliferation [9,165,166,167,168,169,170]. YAP interaction with TEAD transcription factors is essential for CM division, as TEAD1 knockout leads to neonatal lethality [171]. YAP overexpression has been shown to attenuate fibrosis and improve the cardiac function post-MI by inducing CM proliferation [165,166,167,168,169]. Additionally, in crosstalk with the immune system, toll-like receptor 3 (TLR3) activation leads to the inactivation of large tumor suppressor kinase (LATS) and AMP-activated protein kinase (AMPK), thereby activating YAP1 [172].

Several miRNAs play a crucial role in CM proliferation and cardiac repair [173]. miR-590, miR-199a, and members of the miR-17-92 cluster promote CM growth and division, enhancing heart function post-MI and reducing the scar size [174,175,176]. The miR-302-367 cluster represses Hippo signaling, inducing CM proliferation essential for both embryonic development and post-injury cardiac regeneration [177]. Additionally, miR-199a-3p enhances YAP nuclear localization by downregulating its inhibitory kinase, TAO kinase 1 (TAOK1), and the E3 ubiquitin ligase β-TrCP, which mediates YAP degradation [178]. It also inhibits cofilin2, further stabilizing YAP in the nucleus [9]. Another key pathway, the phosphatase and tensin homolog (PTEN)/PI3K/Akt pathway, is activated by miR-301a to drive CM cell cycle re-entry [9,156,176,179].

Other signaling pathways, including the PI3K/Akt/cyclin-dependent kinase (CDK)7, Wnt/β-catenin, platelet-derived growth factor receptor β (PDGFR-β), and Notch pathways, have been implicated in stimulating CM proliferation and promoting cardiac regeneration following heart disease or injury [9,180,181,182,183].

Moreover, the neuregulin/Erbb2/Erbb4 pathway also plays a critical role in CM dedifferentiation and proliferation, contributing to the heart’s regenerative potential [184,185]. Neuregulin 1 (Nrg1) is upregulated after heart injury, and Notch signaling has been linked to increased CM proliferation through Notch1b and DeltaC expression [186,187]. Constitutive activation of the Erbb2 receptor induced binucleated CM proliferation, and constitutive Erbb2 activation (caErbb2) in adult CMs resulted in cardiomegaly with CM hypertrophy, dedifferentiation, and proliferation [9,185,188,189]. These effects were mediated by the ERK, Akt, and GSK3β/β-catenin signaling pathways [9,185,188,189]. Notch signaling regulates the expression of transcripts encoding secreted Wnt antagonists, including Wnt inhibitory factor 1 (Wif1) and Notum1b (palmitoleoyl-protein carboxylesterase b), that in zebrafish (which keep regenerative capacity through adulthood) partially restore CM proliferation in the heart, preventing fibrosis, as demonstrated by comparison with zebrafish without an impaired Wnt pathway [190]. Additionally, ErbB4 has been demonstrated to regulate postnatal CM proliferation in vivo in mouse models [185].

Moreover, genes such as α-SMA, GATA4, Runx1, and DAB adaptor protein 2 (Dab2) are re-expressed in dedifferentiated CMs, potentially facilitating their proliferation and enhancing their multipotent differentiation capacity [151,153,159]. In zebrafish, the inhibition of specific signaling pathways, such as BMP and myocardial NF-κB pathways, has been shown to restrict CM proliferation and regeneration [191,192]. Similarly, blocking GATA4 signaling reduced CM proliferation and impaired their regenerative capacity in zebrafish [193,194].

Targeting cell cycle regulators, such as cyclins and cyclin-dependent kinases (CDKs) promotes CM proliferation; for instance, the overexpression of cyclin D1 activated DNA synthesis in murine CMs [195,196]. Similarly, cyclin D2 overexpression enabled CMs to re-enter the cell cycle and divide, reducing fibrosis and preventing cardiac dysfunction following MI [195,197]. Additionally, cyclin A2 expression induced cardiac hyperplasia by enhancing CM proliferation [198]. Suppressing cyclin-dependent kinase inhibitors (CKIs) has been shown to reactivate the cell cycle, leading to CM proliferation, with evidence of karyokinesis and cytokinesis [199]. Notably, the upregulation of circNfix, a circular RNA, suppressed cyclin A2 and cyclin B1, while increasing miR-214 activity, ultimately promoting CM proliferation and post-injury recovery [199]. miR-128 promotes CM proliferation by targeting SUZ12, which regulates p27, cyclin E, and casein kinase 2 (CK2) [200]. In contrast, miR-195 inhibits CM proliferation and promotes hypertrophy, while inhibiting the miR-15 family enhances CM proliferation post-infarction [152]. Therefore, combining cell cycle activators and inhibitors has been shown to enhance CM division, reduce the scar size, and improve the cardiac function after MI [9,156,195].

Furthermore, alterations in cardiac myocyte metabolism, particularly in response to hypoxia, can also stimulate cell cycle re-entry via hypoxia-inducible factor 1 subunit alpha (HIF1α), further enhancing the heart’s capacity to repair itself [201,202,203]. In murine models, hypoxemia has been shown to reduce mitochondrial oxidative metabolism, decrease ROS production, suppress oxidative DNA damage, and induce cardiac hyperplasia through increased CM mitosis [201,202,203].

Understanding and harnessing these regulatory factors in CM dedifferentiation and proliferation offers promising avenues for developing innovative therapies aimed at repairing damaged cardiac tissue and improving heart function. Further research is needed to refine these mechanisms and enhance their therapeutic potential for cardiac regeneration.

## 5. In Vivo Cardiac Repair: From Reprogramming Techniques to Heart Transplantation

Conventional treatments for MI typically involve medications such as beta-blockers, angiotensin-converting enzyme (ACE) inhibitors, and diuretics, which target various pathways to reduce the oxygen demand and improve cardiac function [83,204]. These treatments help to mitigate sympathetic overstimulation and arrhythmias by managing heart rate and blood pressure, inhibiting the activation of the renin–angiotensin–aldosterone system (RAAS) to prevent adverse cardiac remodeling, and regulating body fluid volume to alleviate symptoms of HF [83,204,205]. In severe cases of post-infarction HF, when pharmacologic therapy proves insufficient, more advanced interventions such as left ventricular assist devices (LVADs) or heart transplantation may be required to replace or support the heart function [83,205]. However, these options are often not viable due to their high costs, limited donor availability, and the complexity of the procedures [204,205].

Cell-based therapies present a promising alternative for cardiac regeneration. However, the field faces significant challenges, including the scarcity of cardiac stem cells (CSCs), the low turnover rate of mature CMs, and difficulties in therapeutic delivery [8,17,206]. Cell reprogramming also shows potential for generating patient-specific CMs, while 3D cardiac patches show promise in tissue repair [8,17,206]. hiPSCs have been extensively studied for their potential ability to differentiate into cardiac cells, however, their application is hindered by safety concerns [17,73]. In contrast, transdifferentiation and dedifferentiation techniques offer a more suitable approach for in vivo cardiac tissue repair by directly reprogramming resident heart cells into new CMs. Despite their advantages, achieving a high reprogramming efficiency in living organisms remains a major challenge [17,73].

**Figure 5:**
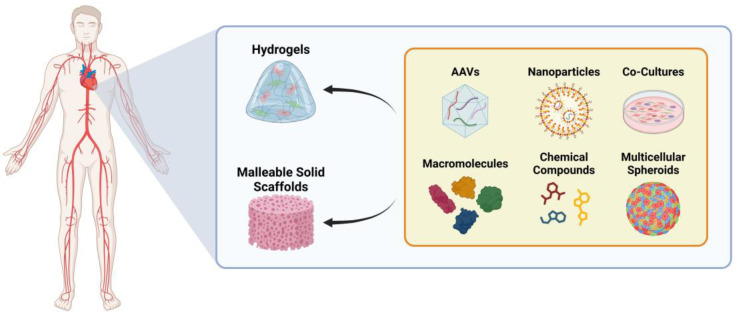
Strategies for the promotion of in vivo cardiac repair. The repair of an injured heart can be promoted by recurring to hydrogels or malleable solid scaffolds embedded with iCPCs, cellular co-cultures (i.e., EC and CM), or pro-angiogenic and growth factors, ultimately, transdifferentiation and dedifferentiation inductors for improved heart regeneration. Created with BioRender.com.

A potential solution to enhance the efficiency of these reprogramming techniques is their integration with 3D cardiac patches, which are available in cellular or non-cellular forms (Figure 5) [17]. Cellular patches are created by seeding live cells into 3D scaffolds. Techniques such as mimetic blood vessel engineering or endothelial cell (EC) co-culture enhance cell survival [8,17,206,207]. Additionally, scaffold modifications with microneedles improve cell integration in the host tissue [208]. Importantly, the field continues to refine the cell growth conditions to enhance the maturation of iPSC-derived CMs on these patches [8,17,143,206,207]. In contrast, non-cellular patches are constructed by incorporating cell derivatives into scaffolds, offering advantages such as greater stability, superior biocompatibility, and reduced tumorigenicity and immunogenicity compared to cellular patches [8,17,143,206,207]. The emerging technology of 3D bioprinting combines biomaterials with various cell types to precisely construct cardiac structures, providing a promising avenue for heart tissue engineering. However, further technological advancements and refinements are necessary before widespread clinical application can be achieved [8,17,143,206,207].

## 6. Ethics and Equity in the Access to Cell Therapies for Heart Disease

The ethical considerations surrounding the clinical use of iPSC-derived cell types are based on three main principles: ensuring the good manufacturing practice (GMP)-compliant generation, cultivation, and differentiation of hiPSCs; evaluating preclinical efficacy and safety; and maintaining ethical and regulatory compliance in stem cell research [209,210]. These cells have versatile applications, ranging from research to potential clinical translation [209,210]. However, ethical concerns arise from the individual genetic fingerprint contained within iPSCs, which could be exploited for detailed genetic research into personal genomes [209,210].

The original iPSC reprogramming technique, which relied on viral vector integration, posed risks of genotoxicity and insertional mutagenesis [209,211]. This led to the development of safer viral vector-non-integrating and non-viral reprogramming methods. Non-viral approaches, such as mRNA, protein, plasmid, and transposon-based methods, offer greater safety but often result in lower efficiency [29,209,211,212]. Despite the well-established CM differentiation protocols, their clinical application remains in its infancy [29,209,210,211,212,213].

One potential solution to reduce immune rejection is to genetically modify a single iPSC line, creating a universal iPSC-based cell therapy, which could significantly lower the costs. In Japan, initiatives such as CiRA’s iPSC bank for regenerative medicine aim to establish a GMP-grade iPSC bank using HLA-homozygous donors to minimize immune rejection [209,210,214,215]. However, this approach does not fully account for the genetic diversity of broader populations, limiting its applicability in many countries.

The concerns surrounding transdifferentiation and dedifferentiation primarily involve the use of lentivirus, which carries a risk of insertional mutagenesis and potential genetic alterations in infected cells [216,217]. This presents a significant safety challenge, emphasizing the need for alternative reprogramming methods to minimize these risks. Additionally, reprogrammed cells may not fully replicate the characteristics of their native cells, raising concerns about their ability to function effectively [65]. Furthermore, the current in vivo studies often provide limited insight into the long-term behavior and integration of reprogrammed cells, underscoring the need for more comprehensive research. Another major hurdle is the low efficiency of transdifferentiation and dedifferentiation, as poor conversion rates lead to prolonged timelines for generating sufficient cells for clinical use. This limitation hinders the timely application of transdifferentiated cells, which is crucial in clinical settings requiring rapid intervention. Addressing these challenges is essential to unlocking the full therapeutic potential of transdifferentiation for treating various medical conditions.

## 7. Conclusions

The current treatments for HF rely on a combination of medications. Whole-organ replacement is the most effective option for end-stage HF but is limited by donor availability and histocompatibility challenges. In response, regenerative medicine offers promising alternatives, including stem cell therapies and reprogramming techniques.

One of these approaches, induced reprogramming to pluripotency, involves generating iPSCs from adult cells to then differentiate CMs for transplantation. However, several challenges remain in terms of improving the efficiency of reprogramming, ensuring the safety of iPSC-derived cells, and achieving controlled differentiation to restore optimal heart function. Ongoing research is focused on addressing these challenges, with the ultimate goal of revolutionizing heart disease treatment through cardiac repair and regeneration. However, while iPSCs hold great promise, their clinical use remains under investigation and subject to rigorous testing and regulatory approval.

Transdifferentiation and dedifferentiation are also promising strategies for inducing high plasticity in terminally differentiated cells, potentially enabling tissue regeneration and repair. Among these approaches, dedifferentiation is considered safer, as it prevents the retrogression to a pluripotent state, thereby reducing tumorigenic risks. To overcome limitations in cell therapy, such as low cell retention, fragility, tumorigenicity, and immunogenicity, cell-derived protein factors, exosomes, and miRNAs have been investigated as potential therapeutic agents.

A key step in optimizing cardiac reprogramming for CVD treatment is understanding CM subtypes and developing cardiac-subtype differentiation protocols. Identifying specific markers and refining the culture conditions may enable the redifferentiation of cells into mature cardiac cell types, enhancing the effectiveness of cardiovascular therapies. Ongoing research focuses on improving the delivery methods for reprogramming factors, targeting specific cell types, and utilizing bioengineered scaffolds to enhance cell integration and function.

Future advancements may need to focus on standardizing reprogramming protocols that enable the ex vivo reprogramming of patient-derived somatic cells within a few weeks, allowing for their expansion and transplantation. A promising approach involves the transplantation of iCPCs embedded in bioengineered scaffolds enriched with pro-angiogenic, growth, and other essential factors. These scaffolds would facilitate tissue regeneration and functional restoration after heart injury. Although an external cell source could help replace the damaged tissue, an alternative strategy involves directly administering scaffolds containing reprogramming factors that promote transdifferentiation and dedifferentiation, along with adjuvants that support tissue regeneration. This approach could enhance cardiac repair efficiency while reducing complications associated with cell transplantation.
