# Correction: Almeida et al. Cell Reprogramming, Transdifferentiation, and Dedifferentiation Approaches for Heart Repair. *Int. J. Mol. Sci.* 2025, *26*, 3063

**DOI:** 10.3390/ijms262210879

**Published:** 2025-11-10

**Authors:** Micael Almeida, José M. Inácio, Carlos M. Vital, Madalena R. Rodrigues, Beatriz C. Araújo, José A. Belo

**Affiliations:** Stem Cells and Development Laboratory, iNOVA4Health, NOVA Medical School|Faculdade de Ciências Médicas, Universidade NOVA de Lisboa, 1169-056 Lisbon, Portugal; micael.almeida@nms.unl.pt (M.A.); carlos.vital@nms.unl.pt (C.M.V.); madalena.rodrigues@nms.unl.pt (M.R.R.);

## 1. Reference Correction

After the publication of the review article [1], one of the referenced manuscripts was found to have been retracted. To enhance the quality of the manuscript, we decided to replace the previous reference number 157 [2] from the original manuscript with the new reference [3] at the same position in the reference list.

Original reference number 157: Zhang, D.; Ning, J.; Ramprasath, T.; Yu, C.; Zheng, X.; Song, P.; Xie, Z.; Zou, M.H. Kynurenine Promotes Neonatal Heart Regeneration by Stimulating Cardiomyocyte Proliferation and Cardiac Angiogenesis. *Nat. Commun.* **2022**, *13*, 6371. https://doi.org/10.1038/s41467-022-33734-7.

New reference number 157: Zhang, X.; Ma, S.; Zhang, R.; Li, S.; Zhu, D.; Han, D.; Li, X.; Li, C.; Yan, W.; Sun, D.; et al. Oncostatin M-induced cardiomyocyte dedifferentiation regulates the progression of diabetic cardiomyopathy through B-Raf/Mek/Erk signaling pathway. *Acta Biochim. Biophys. Sin.* **2016**, *48*, 257–265. https://doi.org/10.1093/abbs/gmv137.

The overall numbering and ordering of the references remain unchanged.

## 2. Text Correction

We have updated the following sentence in the manuscript: “Key pathways involved in the process of dedifferentiation include kynurenine and the aryl hydrocarbon receptor (AHR)–proto-oncogene tyrosine-protein kinase Src (SRC)–yes-associated protein (YAP)/ERK pathway [157]. Kynurenine has been shown to stimulate CM proliferation and angiogenesis via the AHR-SRC-YAP/ERK pathway [157]. Another crucial factor in this process is the inflammatory cytokine oncostatin M (OSM) that activates the Ras/mitogen-activated protein kinase (MEK)/ERK pathway [158–160].”

We have inserted the following corrected paragraph: “Key pathways involved in the process of dedifferentiation include the inflammatory cytokine oncostatin M (OSM) that activates the Ras/mitogen-activated protein kinase (MEK)/ERK pathway [157–160].”

The authors state that the scientific conclusions are unaffected. This correction was approved by the Academic Editor. The original publication has also been updated.
